# Supplementary material for: DMRforPairs: identifying Differentially Methylated Regions between unique samples using array based methylation profiles
Source: BMC Bioinformatics. 2014 May 15;15:141. doi: 10.1186/1471-2105-15-141 (PMC4046028; doi:10.1186/1471-2105-15-141)
Supplement: Additional file 2 — DMRforPairs output for the comparison of A431-MCF7 and NA17018-NA17105. Please start from the HTML files in each folder. Available via the BMC Bioinformatics website. [file 1471-2105-15-141-S2.zip › 1394847754114233_MOESM2_ESM/A431_MCF7/figures/16580.pdf]

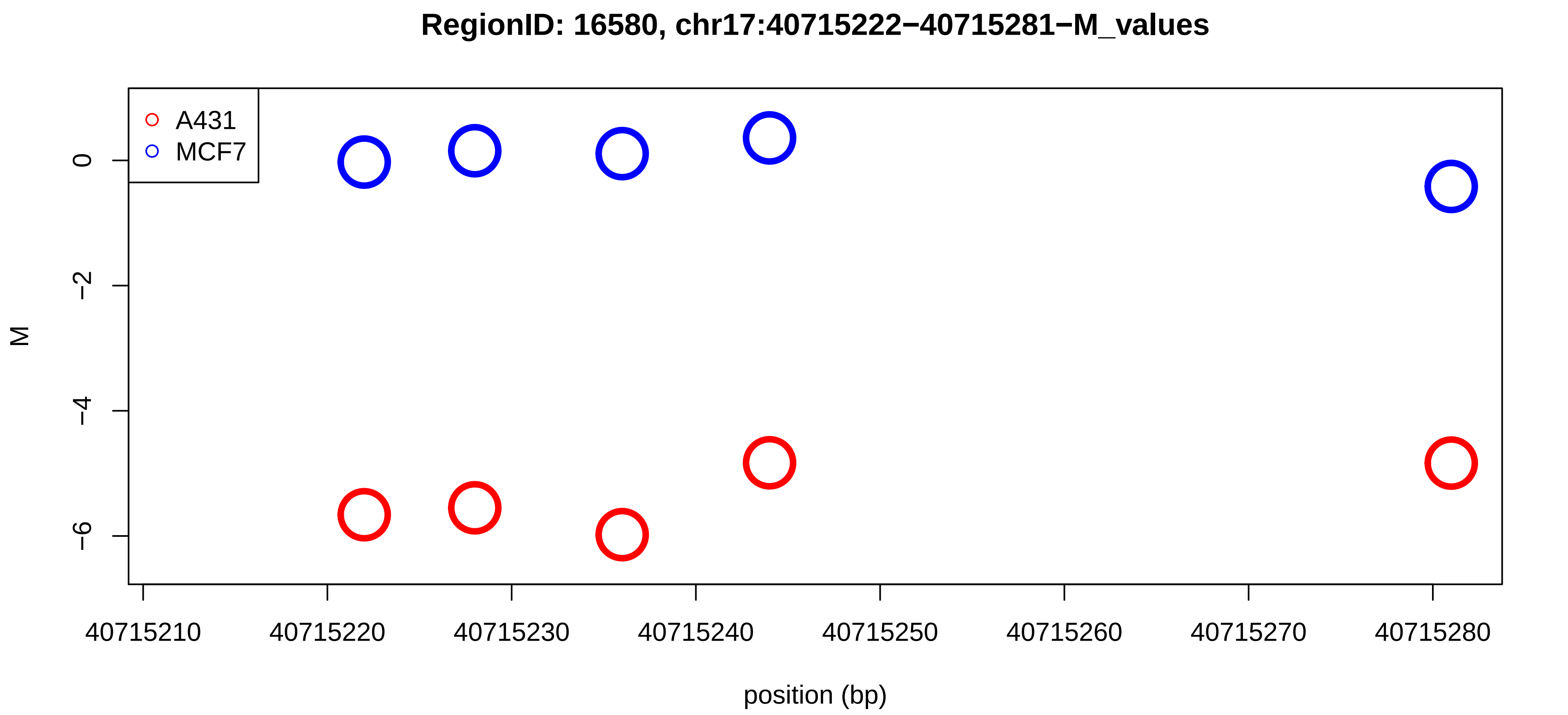
RegionID: 16580, chr17:40715222−40715281−M_values
A431
MCF7
M
0
−2
−4
−6
40715210
40715220
40715230
40715240
40715250
40715260
40715270
40715280
position (bp)

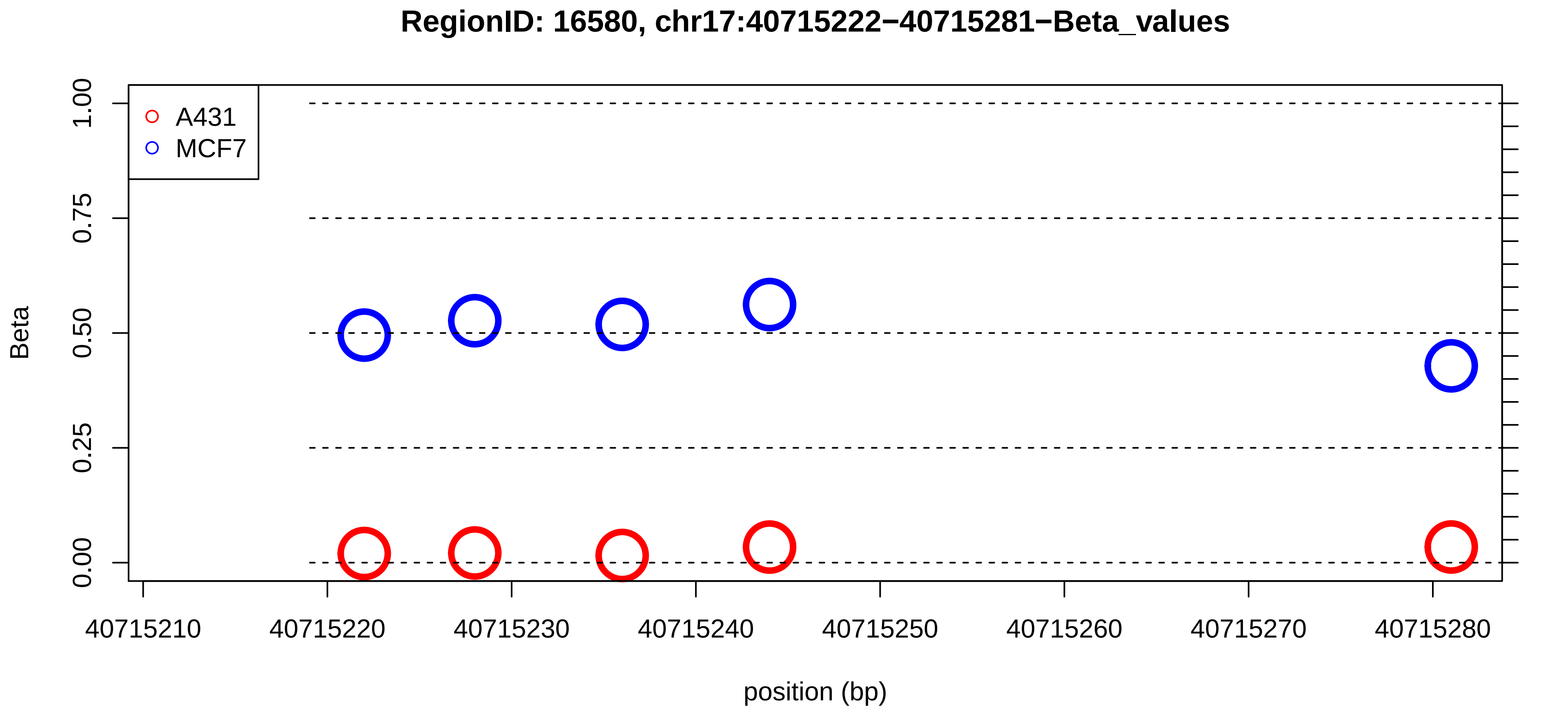
RegionID: 16580, chr17:40715222−40715281−Beta_values
A431
MCF7
Beta
1.00
0.75
0.50
0.25
0.00
40715210
40715220
40715230
40715240
40715250
40715260
40715270
40715280
position (bp)

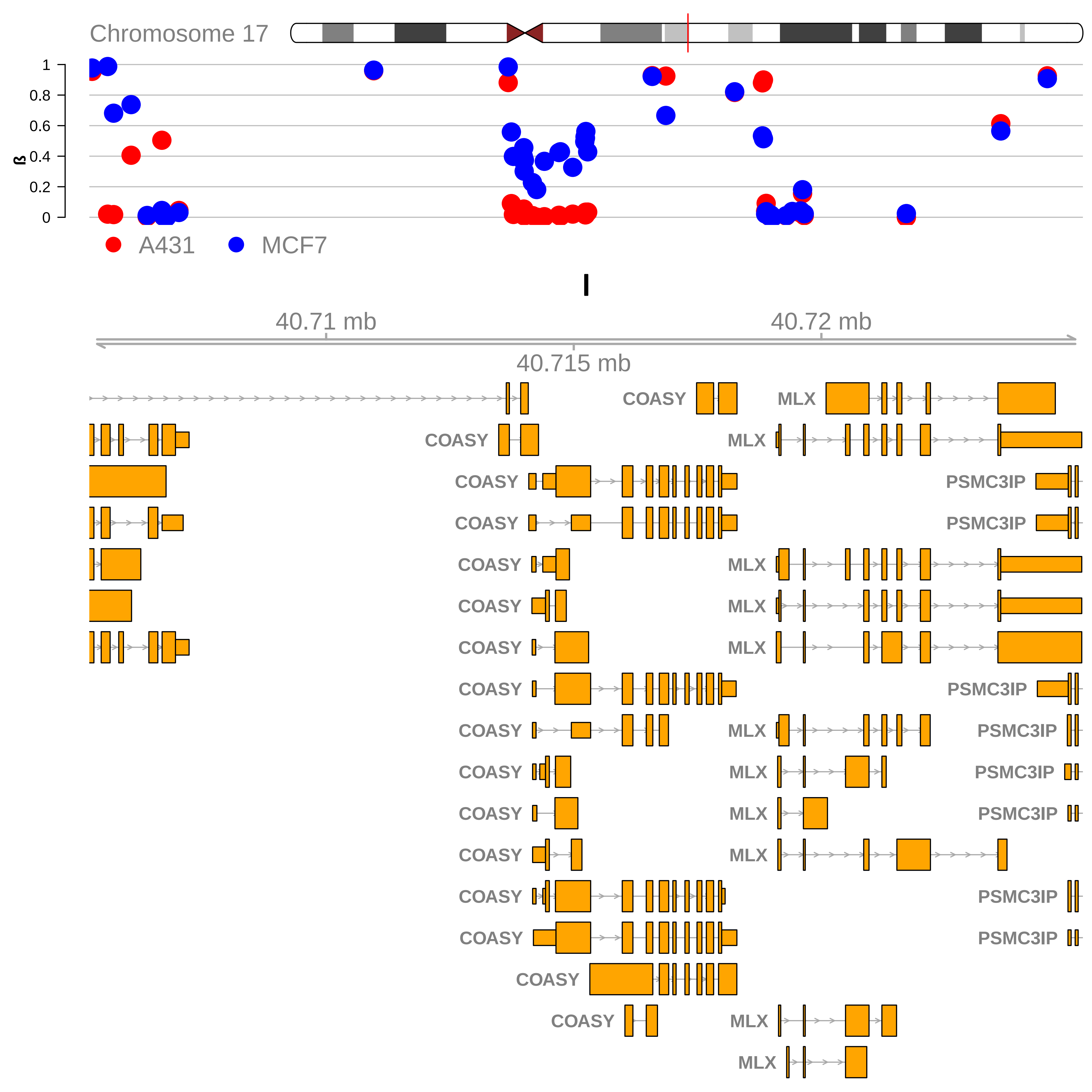
Chromosome 17
ß
1
0.8
0.6
0.4
0.2
0
A431
MCF7
40.71 mb
40.715 mb
40.72 mb
COASY
MLX
PSMC3IP
